# A Blended Intervention for Patients With Knee and Hip Osteoarthritis in the Physical Therapy Practice: Development and a Pilot Study

**DOI:** 10.2196/resprot.5049

**Published:** 2016-02-24

**Authors:** Daniël Bossen, Corelien Kloek, Harm Wouter Snippe, Joost Dekker, Dinny de Bakker, Cindy Veenhof

**Affiliations:** ^1^ Netherlands Institute for Health Services Research Utrecht Netherlands; ^2^ ACHIEVE Centre of Expertise Faculty of Health Amsterdam University of Applied Sciences Amsterdam Netherlands; ^3^ Tranzo Tilburg University Tilburg Netherlands; ^4^ SNIPIT Utrecht Netherlands; ^5^ VU University Medical Center Department of Rehabilitation Medicine VU University Amsterdam Netherlands; ^6^ VU University Medical Center Department of Psychiatry VU University Amsterdam Netherlands; ^7^ University Medical Center Utrecht Department of Rehabilitation, Nursing Science & Sports, Brain Center Rudolf Magnus Utrecht University Utrecht Netherlands

**Keywords:** development, pilot study, osteoarthritis, blended care, eHealth, physical activity

## Abstract

**Background:**

Blended care, a combination of online and face-to-face care, is seen as a promising treatment option. However, actual use of blended interventions in practice is disappointing.

**Objective:**

The objective of this study was two folded. The first aim was to develop a blended exercise therapy intervention for patients with knee and hip osteoarthritis that matches the values of the users and that can be implemented in the daily routine of physical therapists. The second aim was to investigate the feasibility through interviews and a pilot study.

**Methods:**

In this paper, we employed the first 3 steps of the CeHRes road map to develop a blended intervention for patients with knee and hip osteoarthritis. We used interviews, a focus group and discussions with stakeholders to explore the needs, values, and requirements with respect to our to-be-developed blended intervention, which we called e-Exercise. The first version of e-Exercise was tested in a pilot study. Feasibility outcomes, including recruitment rates within each practice, website usage (assignments completed and website visits), and user satisfaction, were measured. In addition, therapists and patients from the pilot study were interviewed to investigate users’ experiences.

**Results:**

The study captured important information about stakeholders’ needs and perspectives. Based on our findings, we created a first version and attuned the application’s content, functionality, and structure. Patients and, to lesser extent, physical therapists were satisfied with the e-Exercise intervention. Eight patients were recruited by 8 physical therapists. Of the 8 patients, 6 completed more than 7 of 12 modules.

**Conclusions:**

This study outlines the development and feasibility of a blended exercise therapy intervention for patients with knee and hip osteoarthritis. E-Exercise offers an alternative approach in the physical therapy treatment of knee and hip osteoarthritis. This study provides valuable information to conduct a further trial to evaluate the (cost) effectiveness of e-Exercise compared to usual physical therapy.

**Trial Registration:**

Netherlands Trial Register Number: NTR4224; www.trialregister.nl/trialreg/admin/rctview.asp?TC=4224 (Archived by WebCite at http://www.webcitation.org/6fOK4lrTO).

## Introduction

Knee and hip osteoarthritis (OA) are leading causes of disability in older people [1]. In the upcoming years, the number of people with knee and hip OA will grow due to the aging population and escalating risk factors, such as obesity [2]. Since there is no cure for OA, exercise, education, and medication are considered to be cornerstones of its treatment [3,4].

Although patients with knee and hip OA generally tend to avoid physical activity [5], physical exercise is one of the most effective and recommended treatment modalities [3,4]. Exercise therapy, generally provided by a physical therapist, is a regimen of physical activities with the aim to change patients’ lifestyle behavior and improve patients’ overall function [6]. Therapeutic exercise therapy consists of strengthening, aerobic, flexibility, and/or functional exercises. Multiple studies have demonstrated the beneficial effects of exercise therapy in patients with knee and hip OA. Exercise therapy has positive effects on pain perception and self-reported physical function [7,8]. However, therapeutic exercise therapy is labor-intensive, costly, and often not covered by the health insurance, especially over the long term. So, although helpful, physical therapy is not accessible for many OA patients. According to current estimates, only 7% of all patients with knee and hip OA who are seen in general practice are actually referred to a physical therapist [9].

There is a clear need for more feasible and easily accessible strategies in order to regulate therapeutic costs and make exercise therapy attainable for a broader range of OA patients. This can be accomplished through self-management support. Self-management implies individuals’ ability to manage the symptoms, treatment, physical and psychosocial consequences, and lifestyle changes inherent in living with a chronic disease [10]. The use of eHealth has the potential to support self-management in patients with OA treatment beyond the walls of the physical therapy practice. Examples include, but are not limited to, Web-based interventions and mobile health interventions that can help to improve patients’ health behavior and corresponding health outcomes [11,12]. The 24/7 availability of information may improve treatment compliance, which is critical for the success of physical therapy [13]. Moreover, embedding eHealth within daily practice also has the potential to substitute a part of the face-to-face contacts and alleviate the pressure on health services. Furthermore, eHealth opens up new avenues to reach new patient groups, especially for those who have minimal or no coverage for physical therapy expenses.

Although promising in terms of evidence and accessibility, the adoption of eHealth technologies is disappointing [14]. Embedding eHealth technologies in daily practice is a complex and time-consuming process, more than initially anticipated [15]. So far, eHealth interventions are primarily used outside the health care setting and rarely integrated as part of the treatment. To illustrate, only 1% of all patients in the physical therapy practice use therapeutic-provided eHealth interventions, such as online self-management treatments or online exercises [16]. The uptake and implementation of eHealth innovations in practice is dependent on various factors that can be broadly divided into 4 categories: (1) characteristics of the technology itself, such as ease-of-use and quality of the intervention; (2) characteristics of the end-users, such as perceived usefulness, perceived support from family/colleagues, skills, and knowledge; (3) characteristics of the organization, such as formal endorsement and costs; and (4) policy and legislation, such as privacy issues and reimbursement schemes for eHealth services [17].

The success of eHealth is hampered by insufficient attention paid to abovementioned determinants during the development process. The majority of eHealth technologies is created through ad-hoc procedures without a thoughtful approach [18]. High rates of non-usage and implementation difficulties are a normative phenomenon in eHealth [9,14,19]. The peripheral position of end-users and inadequate input of stakeholders lead to a mismatch between technology and context, which explains why eHealth does not reach its full potential in practice [20]. The involvement of stakeholders—such as health care providers, policy makers, and health insurers—provides direction for the development of eHealth technologies. Co-creation, the engagement of users and other stakeholders throughout the development process, is an important strategy in order to meet the values and needs of stakeholders.

**Figure 1:**
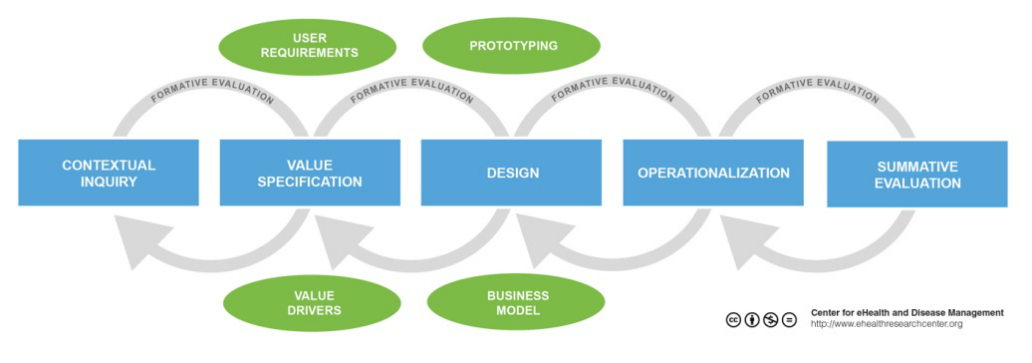
CeHRes road map.

The Centre for eHealth Research and Disease Management (CeHRes) road map is a development approach in which co-creation plays a central role [20]. This CeHRes road map anticipates the needs and values of stakeholders and consists of 5 steps (Figure 1). In this article, we employed the CeHRes road map to develop a new blended intervention for patients with knee and/or hip OA. This intervention, which will be a combination of eHealth and face-to-face care, will be integrated into daily physical therapy practice. This proposed program aims to promote a physically active lifestyle among patients with knee and hip OA. The objective of this study was 2-folded. The first aim of this study is to develop a human-centered eHealth physical activity intervention that matches the values of users and that can be implemented in the daily routine of physical therapists. The second aim was to investigate the feasibility through interviews and a pilot study. To our knowledge this is the first study investigating a blended exercise therapy intervention for physical therapists.

## Methods and Results

In order to enhance clarity and optimize the execution of each step, we have chosen to present the methods and results sections together. In the following section, we describe the first 3 steps of the CeHRes road map, namely the contextual inquiry, value specification, and design. We present a pilot study on the feasibility of the blended intervention. The first 3 stages of the CeHRes road map and pilot study provide the basis for steps 4 (operationalization) and 5 (summative evaluation), which will be conducted in a later phase of the project. The study has been approved by the Medical Ethical Committee of the St. Elisabeth hospital Tilburg, the Netherlands (Dutch Trial Register NTR4224).

### Contextual Inquiry and Value Specification

#### Methods

**Figure 2:**
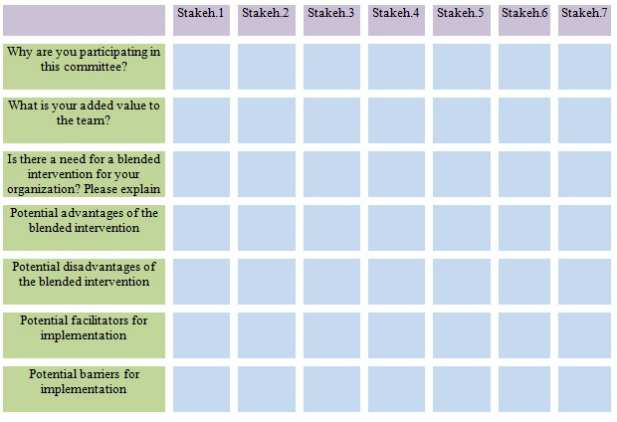
Stakeholders’ needs and perspectives.

During the contextual inquiry and value specification we aimed to establish stakeholders’ most important needs, values, and requirements with respect to our to-be-developed blended intervention. The input of this phase was mainly based on another project, executed by the same authors [21]. In this previous work, we developed and evaluated the Web-based intervention Join2move. Join2move is a self-guided intervention and contains automatic functions without human support. The 9-week program is directed at increasing the level of physical activities in a time-contingent manner (fixed time points). More information about the intervention and used methods can be found elsewhere [21]. For the development of our blended intervention, e-Exercise, a focus group among 7 physical therapists was conducted. Physical therapists, who had an extensive experience in the field of OA, were recruited through the website of The Royal Dutch Society for Physical Therapy to participate. The focus group was facilitated by 2 moderators (CK and DB) and lasted approximately 120 minutes. During the focus group, we used a topic guide that contained questions related to the content needs of the intervention, reimbursement, and frequency of face-to-face contact. The focus group discussion was audio-recorded and subsequently summarized. Summarized texts were subsequently read and discussed between 2 reviewers (DB and CK) to gain an overall understanding of the needs and perspectives with respect to the e-Exercise intervention. Furthermore, an implementation committee was formed with different stakeholders. The stakeholder committee consisted of patients with knee and/or hip OA, the Royal Dutch Society for Physical Therapy, 2 rehabilitation centers, the Dutch arthritis foundation, an eHealth entrepreneur, and a health insurer. The committee meetings were held 3 times and were led by the last author (CV). At each meeting, stakeholder members were encouraged to discuss and share their thoughts about the development and implementation process of the blended intervention. The results from these discussions provided direction for further development of the blended intervention. We created a matrix in order to summarize and analyze needs and perspectives of the individual committee members (Figure 2).

#### Results

Physical therapists in the focus group indicated that a blended intervention will be a useful instrument in the treatment of OA patients. The 24/7 availability of information and exercises, the possibility to extend the physical therapy treatment in the home environment of the patient, and the potential to enhance the adherence of home exercises were mentioned as possible advantages. On the other hand, the fact that the proposed blended intervention aims to substitute conventional visits may lead to reduced revenues per patient. According to physical therapists, this lack of financial incentive was seen as a potential barrier to use the proposed intervention in practice. The results in the matrix, which represent the stakeholders’ needs and perspectives with respect to Join2move, showed positive attitudes toward the to-be-developed blended intervention. As a stakeholder from a rehabilitation institute stated: “Patients will benefit from the blended intervention because it is cheap, independent of time or place, and promotes self-management in the home environment of OA patients.” Another facilitator for implementation is the potential to reduce treatment costs. An employee of a health insurance company summarized this by saying: “The proposed blended intervention will possibly result in lower costs since the average number of sessions will be decreased. This will lead to a cost reduction of the OA treatment.” The patients were also positive. They had a positive attitude toward the idea that eHealth will be an integrated part of their treatment, especially for information and education purposes.

### Design

#### Methods

E-Exercise is a combination of (1) visits with a physical therapist, and (2) a Web-based physical activity intervention. The technical functionality of the Web-based part is based on a previously developed physical activity intervention [22]. This initial Web-based intervention contained only self-directed features without the integration of physical therapy sessions. To investigate whether and how the initial Web-based intervention fits the day-to-day requirements and routines of physical therapists, different content scenarios were presented during a second focus group session with physical therapists. These scenarios concentrated on several themes, such as the number of face-to-face visits, extent of (online) interaction between patient and physical therapist, and website content such as videos and design and education topics. Results were used to change the first Web-based intervention and create the blended intervention e-Exercise. This first blended version of e-Exercise was then tested in a pilot study.

#### Results

**Figure 3:**
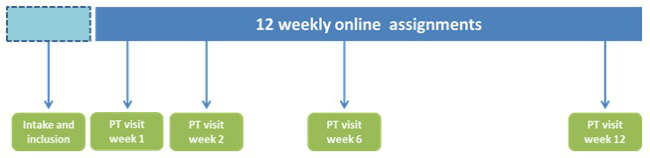
Overview of the 12-week e-Exercise treatment.

**Figure 4:**
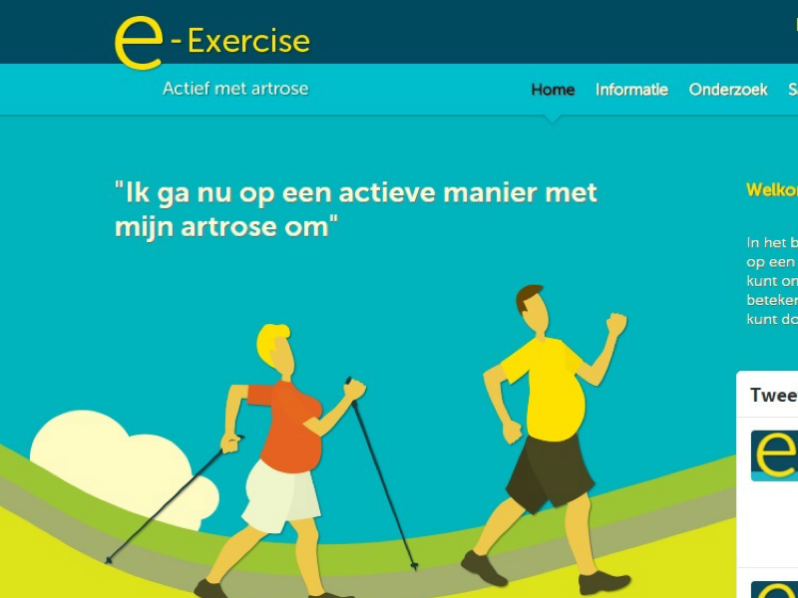
The e-Exercise home page.

Over the course of a half year, a team of experts from NIVEL developed the e-Exercise program. The starting point of the development process was a previously developed Web-based exercise intervention [23] and the Dutch guidelines for physical therapists [24]. The intervention is delivered over a period of 12 weeks. During the 12 weeks, patients receive 4 face-to-face sessions with a physical therapist and are supposed to complete 12 online assignments (Figure 3). The physical therapists were encouraged to follow a fixed treatment protocol. The website has a portal for both patients and physical therapists and contains text- and video-based information. The core element of the website activities is the promotion of moderate physical activities, such as cycling, walking, or swimming in the home environment of patients. Every week, automatic generated physical activity exercises are posted on a password-secured website in which a self-chosen physical activity is gradually increased in a time contingent manner (ie, fixed time points). Time-contingency means that physical activities are increased on fixed time quotas rather than guided by OA-related symptoms such as pain and fatigue. This strategy is derived from the behavioral graded activity intervention and concepts of operant conditioning [25]. The e-Exercise home page is shown in Figure 4. Illustrative screenshots of the e-Exercise website are presented in Multimedia Appendix 1.

### Pilot Study

#### Methods

##### Study Design and Objective

This pilot study employed a multicenter 1-group design. The purpose was to evaluate the feasibility of the e-Exercise treatment in the daily practice of physical therapists.

##### Procedures and Participants

Physical therapists working in a private practice were recruited through the website of the Royal Dutch Society for Physical Therapy and the social network of the authors. Eventually, 8 physical therapists were included in the pilot study. All participating physical therapists received a half day of training about the study procedures and how to use e-Exercise in their practice. Eligible patients, who visited a participating center during the study period, were enrolled by the physical therapists. Enrollment started on March 3, 2014, and ended May 6, 2014. Participants were suitable for inclusion if they (1) were aged 40 to 80 years and (2) had the diagnosis OA of the knee and/or hip according to the clinical criteria of the American College of Rheumatology [26]. Participants were not suitable if they (1) were on a waiting list for a hip or knee replacement surgery, (2) had contraindications for physical activity without supervision, (3) had a physically active lifestyle, (4) participated in a physical therapy for OA and/or physical activity program in the last 6 months, (5) had no access to the Internet, and (6) were unable to understand the Dutch language. Interested patients who were willing to participate and met the eligibility criteria were sent an information letter about the study and an informed consent form. Once written informed consent was obtained, participants were invited to fill out an online baseline questionnaire. After baseline completion, participants were included in the study.

##### Feasibility

Feasibility measures included website usage, user satisfaction with the website, and recruitment rates of participants within each practice. Program use was measured by the number of modules completed. Based on a previous study [21], we considered the completion of 7 out of 12 modules as feasible. User satisfaction was measured through the System Usability Scale (SUS) [27]. For this development study, an SUS score of 51 points or more was considered feasible [28]. Moreover, participating physical therapists and participants were invited for interviews to learn about their experiences with e-Exercise. Semistructured interviews were conducted on a subsample of 5 physical therapists and 4 patients. Interviews were audio-recorded and transcribed with interviewee’s permission. An interview guide with open questions was employed to provide structure to the interviews (see Multimedia Appendix 2). Transcribed texts were read and thematic trend analysis was conducted to identify, analyze, and report recurrent patterns. Themes were discussed by CK and DB to gain an overall understanding of the usability and user satisfaction.

#### Results

##### Feasibility

A total of 8 eligible OA patients were included in the pilot study by the 9 participating physical therapists. Patients were on average 62 years old, had 1 or more comorbidities (88%), and most of them were female (75%). None of the participants withdrew from the study. An overview of the sample characteristics is presented in Table 1. Overall, patients and, to a lesser extent, physical therapists were satisfied with the e-Exercise intervention. Results from the system usability scale among patients revealed an average score of 79 points (SD 8.7) on a 100-point scale questionnaire, which can be considered as a good score [28]. Usability scores from physical therapists were considerably lower, namely 64 (SD 7.7). This rating can be interpreted as “fairly” good [28]. Login-analyses showed that 6 out of the 8 patients completed more than 7 of the 12 modules. Over the 12-week intervention period, patients visited the website 33 times on average. Prior the study, we intended to recruit 2 patients per participating physical therapist. However, during the 10-week enrollment period, only 5 of the 8 physical therapists recruited 8 patients in total. Physical therapists reported that e-Exercise is only suitable for a small subset of patients. Most of the patients with knee and hip OA prefer traditional face-to-face treatments over the blended intervention or did not meet the study inclusion criteria. One physical therapist said: “Most of the patients with knee or hip osteoarthritis that I have seen were not interested in participating in e-Exercise because they preferred face-to-face guidance. Other patients did not have a computer or had an already physically active lifestyle.”

Overall, interviewees were satisfied with the intervention. One patient summarized this sentiment by saying: “I have told many friends and family that this is a great program because the program motivates you to perform exercises in your own time. I would therefore definitely recommend e-Exercise to others.” Physical therapists also expressed positive feedback regarding the content of e-Exercise. To cite 1 therapist: “I am especially pleased with the information about osteoarthritis provided by the videos. More insight into the disease and the role of pain is important prerequisite to encourage a physically active lifestyle.” Although physical therapists were generally satisfied, they stressed that e-Exercise must be adapted for suitable integration into practice. As 1 physical therapist commented: “The program provides no insight [into] which modules patients receive. This was truly a downside of the program because I had little or no control over patients’ progress.” It was also reported by some patients that they liked the effective approach of e-Exercise. One patient commented: “I liked the effective approach of the intervention. You need only a few face-to-face treatments to get on track. The provision of weekly physical therapy sessions is not useful because you have to exercise yourself.”

**Table 1:** Baseline demographics and characteristics OA patients.

Participants (N=8)		
**Gender, n (%)**		
	Female	6 (75)
	Male	2 (25)
Mean age, y (SD)		61.88 (14.53)
**Location OA, n**		
	Knee	4
	Hip	3
	Both	1
**Duration of symptoms, n**		
	< 1 year	2
	1-3 years	1
	3-7 years	2
	≥7 years	3
**Education, n**		
	Low	2
	Middle	2
	High	4
**Comorbidity, n**		
	None	1
	1	4
	≥2	3

## Discussion

### Principal Findings

Provision of blended care requires a harmonious integration of technology into practice, combining complementary face-to-face treatments with eHealth technology. Implementing a blended intervention into health care is a complex process that changes existing routines, relationships, and budgets. Developers and researchers have to anticipate these implementation difficulties. While research supports the effectiveness of health technology, health care professionals often lack the time, skills, and resources to integrate eHealth into their daily practice. Input of end-users and other stakeholders throughout the development process is a prerequisite for the successful implementation of blended interventions into practice [20]. The aim of this study was to develop and investigate the feasibility of a blended exercise therapy intervention for patients with knee and hip OA that can be implemented in the daily routine of physical therapists.

The involvement of patients, physical therapists, and other stakeholders was extremely valuable throughout the development process. The first 3 phases of the CeHRes road map yielded unique insights into different needs and values of end-users and various stakeholders. Steps from the CeHRes model were not purely sequentially executed but involved a continuous process. For instance, the identification of needs and problems was mainly derived from experiences with a previous eHealth project, rather than a separate phase in the current project. The results from the post-pilot interviews demonstrated that e-Exercise is feasible in the treatment of patients with knee and hip OA. In line with the findings from the study by Pietrzak et al [29], participants were positive toward the use of eHealth in the treatment of OA. Users considered the usability of e-Exercise as “good.” Interviews with physical therapists and patients revealed a beneficial impact on the organization process of care. The possibility to stimulate exercises in the home environment and to enhance exercise adherence were cited as major advantages. However, the inability to monitor patients’ progress between consultations seemed to be a drawback. Monitoring was therefore added in the latest version of e-Exercise. Another sign that demonstrated the feasibility of e-Exercise were the usage rates. Of the 8 participants, 6 completed more than 7 of the 12 modules. These usage rates can be considered reasonably high when compared with a previous study [21].

The visit-based method of recruitment was challenging. Over the 10-week enrollment period, we intended to recruit 2 patients per participating physical therapist. However, only 8 eligible patients were recruited by 8 physical therapists. Others have reported similar challenges with the recruitment of patients [30-32]. The lack of remuneration may have contributed to the disappointing recruitment rates since there is no financial incentive to adopt e-Exercise in practice. On the contrary, the use of e-Exercise might even lead to reduced revenues per patient. Another possible explanation for the poor recruitment rates is the small pool of eligible OA patients. Primary care data from the Netherlands shows that only 2% of all patients seen in the physical therapy practice have OA [33]. General practitioners, the gatekeepers of the Dutch health care system, have a strong influence on the influx of patients into physical therapy setting. General practitioners should therefore be informed of the availability and possibilities of blended interventions. This might influence the referral behavior of general practitioners positively.

### Limitations

The findings of the pilot study need to be interpreted in light of several limitations. The small number of participants and the absence of a control group are major limitations of the current study. Moreover, the generalizability might be limited by the self-selected sample in this study. Obviously, included physical therapists are techno-enthusiasts who are more willing to adopt technology in their practices than are others.

### Conclusions

Results from this study are valuable to set up a follow-up study to compare e-Exercise with usual physical therapy. We plan to conduct a larger, adequately powered, randomized controlled trial to investigate the effectiveness (including cost effectiveness) of e-Exercise [34]. The recruitment of patients was a true challenge in this study. We therefore need to pay extra attention to the recruitment process and find additional avenues to increase recruitment rates for the randomized controlled trial. Given the 1:1 recruitment ratio of this pilot study, we aim to recruit at least 200 physical therapists. We also plan to use strategies to encourage physical therapists to include more participants, to engage general practitioners in the recruitment of patients, and to extend the inclusion period of patients.

## Appendix

Multimedia Appendix 1 Screenshots website.

Multimedia Appendix 2 Interview guide.

## Abbreviations

CeHRes: Centre for eHealth Research and Disease management
OA: osteoarthritis
SUS: System Usability Scale
