# Supplementary material for: A Blended Intervention for Patients With Knee and Hip Osteoarthritis in the Physical Therapy Practice: Development and a Pilot Study
Source: JMIR Res Protoc. 2016 Feb 24;5(1):e32. doi: 10.2196/resprot.5049 (PMC4785239; doi:10.2196/resprot.5049)
Supplement: Multimedia Appendix 1 [file resprot_v5i1e32_app1.pdf]

Weekoverzicht

1. basisniveau 20 minuten
2.
3.
4.
5.
6.
7.
8.
9.
10.
11.
12. (korte-termijn)doel 45 minuten

Activiteit | Oefeningen | Informatie | Info-theek | Oefen-theek

Evaluatie van de vorige week

De beweegopdracht van vorige week was:
Beweegopdracht 1: 35 minuten fietsen
Beweegopdracht 2: 35 minuten fietsen
Beweegopdracht 3: 35 minuten fietsen

Kunt u aangeven in hoeverre u deze opdracht heeft uitgevoerd?
- Ik heb precies gedaan wat in de opdracht stond aangegeven
- Ik heb minder gedaan wat in de opdracht stond aangegeven
- Ik heb meer gedaan wat in de opdracht stond aangegeven

Hoeveel pijn heeft u gemiddeld ervaren in de afgelopen week? (0=geen pijn, 10=ondragelijke pijn)

0 1 2 3 4 5 6 7 8 9 10

Evaluatie opslaan

De opdracht van deze week

Doe op drie verschillende dagen van de week één van de onderstaande beweegopdrachten. Wij adviseren u om de beweegopdrachten te verspreiden over de week, zo voorkomt u overbelasting.
Beweegopdracht 1: 35 minuten fietsen
Beweegopdracht 2: 35 minuten fietsen
Beweegopdracht 3: 40 minuten fietsen

Voor uw oefeningen van deze week verwijzen we u naar het tabblad Oefeningen. Onder het tabblad Informatie komt u deze week meer te weten over Beweegplan maken.

e-Exercise

Log hier uit

Actief met artrose

Fysiotherapeut Admin Pagina

Patiënt | Activiteit | Oefening

Info-theek | Oefen-theek

- Home
- Mijn patiënten
- Een patiënt aanmelden
- Mijn profiel
- Handleiding
- FAQ

Activiteit

Centrale activiteit: fietsen

Opbouw van het programma

week	training 1	training 2	training 3	evaluatie	pijn score (0 - 10) (0= geen pijn, 10= ondraaglijke pijn)
2	10 min.	10 min.	10 min.	exact	4
3	15 min.	15 min.	15 min.		
4	15 min.	20 min.	20 min.	more	3
5	20 min.	20 min.	20 min.	more	1
6	25 min.	25 min.	25 min.		
7	25 min.	30 min.	30 min.	exact	4
8	30 min.	30 min.	30 min.		
9	35 min.	35 min.	35 min.		
10	35 min.	35 min.	40 min.		
11	40 min.	40 min.	40 min.		
12	45 min.	45 min.	45 min.		

Wat is artrose?

Artrose is een veel voorkomende reumatische gewrichtsaandoening waarbij de kwaliteit van het kraakbeen tussen twee gewrichten vermindert. Daarnaast wordt de hoeveelheid kraakbeen minder, waardoor botstukken dichter bij elkaar komen te liggen.
Kraakbeen is een gladde bekleding tussen twee botten en kan worden gezien als de schokdemper van onze gewrichten. Als het kraakbeen dunner wordt, neemt de druk op het bot toe. Hierdoor kunnen er knobbels op het bot ontstaan en soms kan het omliggende weefsel (het gewrichtskapsel) ontstoken raken. Artrose kan in elk gewricht voorkomen, maar in de knie en heup komt artrose het meest voor.

U kunt gewrichten vergelijken met een scharnier. Het laagje glad kraakbeen zorgt ervoor dat het gewricht gemakkelijker kan buigen en strekken. Ook de vloeistof rondom het gewricht (synovia) zorgt ervoor dat botten makkelijk langs elkaar glijden. U kunt zich voorstellen dat door de artrose het gewricht minder soepel loopt.

In het onderstaande plaatje ziet uw links een gezond gewricht en rechts een gewricht dat is aangedaan door artrose. De kraakbeenkussentjes zijn bijna helemaal weg. Het bot probeert de schade te herstellen en wordt daardoor dikker en breder. Aan de rand ontstaan knobbels (osteofyten).

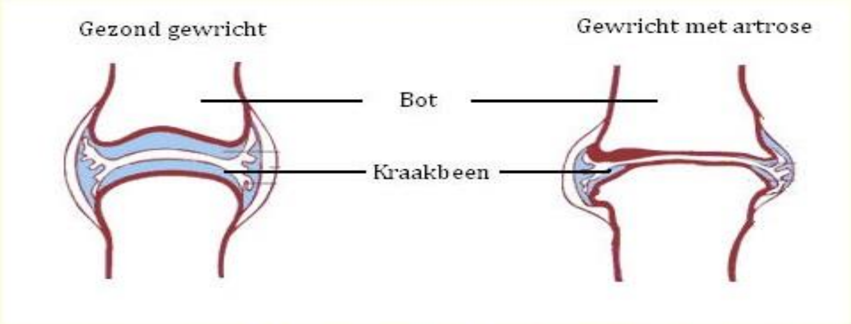

Artrose is een chronische aandoening die in de volksmond vaak "gewrichtsslijtage" wordt genoemd. Het is belangrijk om te weten dat de gewrichten niet slijten, maar dat de kwaliteit minder wordt. Bij het woord "slijtage" wordt namelijk vaak gedacht dat er niets aan te doen is. Artrose zal niet meer overgaan, maar tijdens dit

Patiënt

Naam: Jan Janssen (corelien@hotmail.com)
Laatste login datum: 18 aug 2015
Aatal keren gebruikt: 14 keer
In week: 10

Resultaten basismeting

Datum	Aantal minuten		Pijn tijdens activiteit: van 0-10 (0= geen pijn, 10= ondraaglijke pijn)	
12-06-2015	20	min.	5	Dag 1 opslaan
14-06-2015	25	min.	4	Dag 2 opslaan
16-06-2015	15	min.	7	Dag 3 opslaan

Doelen bepalen

Selecteer voor deze patiënt het korte termijndoel. Het doel dient hoger te zijn dan het huidige niveau. Het huidige niveau is gemiddeld 20 minuten fietsen per sessie

Over 12 weken wil de patiënt 45 minuten kunnen fietsen.

Het lange termijn doel van de patiënt is
Op de fiets naar kleindochter in Bunnik en weer terug (totaal 20km)

Start het beweegprogramma voor Jan Janssen | Wijzig doelen

Beweegplan

e-Exercise

Actief met artrose

Hallo Jan Janssen | Mijn profiel | Log hier uit

Informatie Onderzoek Samenwerking Contact Mijn e-Exercise

Oefen-theek

- Bovenbeenspieren aanspannen
- Beenstrekkers
- Dijbeenspieren
- Kniebuigingen
- Uitvalspas
- Bilspieren
- Hak naar de bil
- Kuitspieren
- Opstaan uit de stoel
- Evenwicht op 1 been
- Bruggetje
- Binnenkant beenspieren rekken
- Bilspier rekken

Activiteit Oefeningen Informatie Info-theek Oefen-theek

Bilspieren

Ga rechtop achter een stoel of tafel staan. Beweeg uw been, met gestrekte knie, naar achteren en houdt deze houding een drietal seconden vast. Breng vervolgens het been terug.

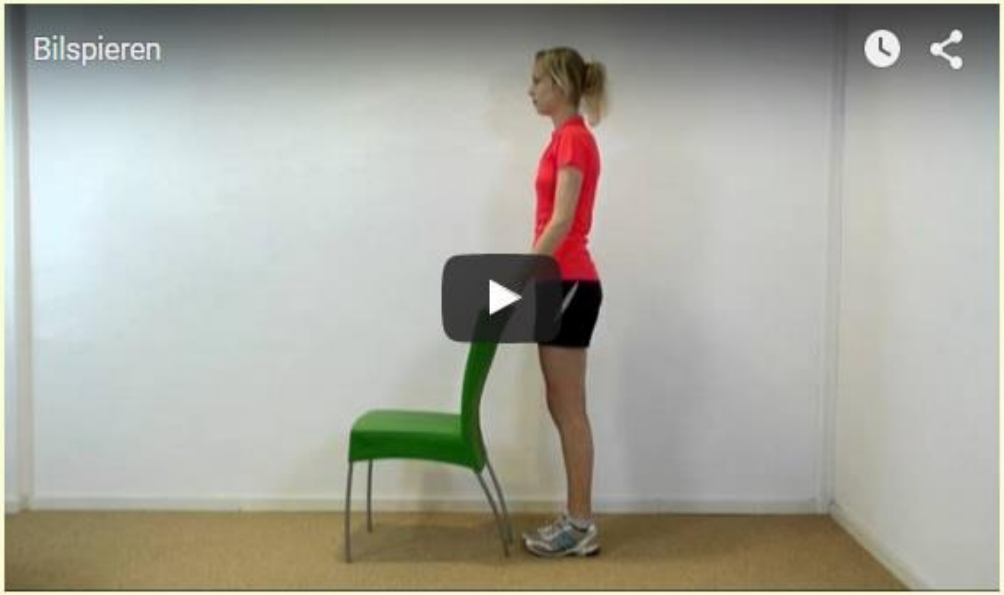

For more information, go to: https://www.youtube.com/watch?v=4l9GoQWWy58
