# Supplementary material for: A Blended Intervention for Patients With Knee and Hip Osteoarthritis in the Physical Therapy Practice: Development and a Pilot Study
Source: JMIR Res Protoc. 2016 Feb 24;5(1):e32. doi: 10.2196/resprot.5049 (PMC4785239; doi:10.2196/resprot.5049)
Supplement: Multimedia Appendix 2 [file resprot_v5i1e32_app2.pdf]

Appendix 1: Interview guide

- Introduction of ourselves, instruction and aim of the interview and general information about the project
- Asking permission for audio recording
- Providing information about privacy policy

- What was your motivation to participate in the e-Exercise study?
    - Insurance coverage, advantages of the web-based intervention (patients)
- Information on the website
    - Reasons for logging-in, how many logging in, study information, information about OA and related topics etc. (patients and PT's)
    - What is your opinion about the information on the website? Useful? (patients and PT's)
    - What did your learn from the website? Also with respect to health behavior. (patients)
- Why have you chosen to participate in this program/study? (patients and PT's)
- What are your experiences with the program (patients and PT's)
    - Advantages/ Disadvantages
    - Language
    - Tone of texts
    - Did you experience difficulties?
    - Did you use help from others?
    - Will you recommend e-Exercise to others?
- What is your opinion about the content of the program? (patients and PT's)
    - Which elements did you (not) like?
    - Are there elements of the program that must be improved?
    - Flexibility of the program to tailor on needs, progress and physical capacity etc. (PT's only)
    - Video exercises
    - Information and themes
- Appropriateness of the amount of face-to-face guidance (patients and PT's)
    - In order to reach the treatment goals
- Overall use and familiarity with technology (patients and PT's)
    - Use of technology in practice/in the treatment of patients, such as digital agenda, YouTube videos, digital questionnaires etc.
- Did you participate in a physical therapy intervention before? (patients)
    - If yes, what are the main differences?
- Were other people aware/involved during participation? (patients)
    - What was the opinion of family/friends/neighbors about your participation in e-Exercise?
    - Did you receive positive/negative reactions from others?
    - Did people notice that you performed more physical exercises?
-What were the patient reactions with respect to e-Exercise? (PT's)
    - Positive and/or negative?
    - Do you think that patients benefit from de e-Exercise intervention?
    - Does e-Exercise acts as a form of publicity in order to reach new target groups?
- How many patients were or were not suitable to participate the study? (PT's)
    - What were the reasons for in- and exclusion?
    - What is the main reasons why patients are or are not interested in e-Exercise?
